# 20201 Validating an in-car telemetry system for detecting frequency and severity of driving errors in patients with glaucoma.

**DOI:** 10.1017/cts.2021.479

**Published:** 2021-03-31

**Authors:** Sharon Sabapathypillai, Monica Perlmutter, Manik Goel, Bradley Wilsone Gordon, Anjali Bhorade

**Affiliations:** Washington University-Saint Louis

## Abstract

ABSTRACT IMPACT: The car telemetry system may be an ideal method to accurately and reliably evaluate and compare at-risk driving errors between older drivers with and without glaucoma. OBJECTIVES/GOALS: Our project aims to determine whether an in-car telemetry system used during an on-road driving evaluation can accurately and reliably evaluate driving errors in lane maintenance and visual scanning and objectively quantify the frequency and severity of these errors in glaucoma patients. METHODS/STUDY POPULATION: This is a single center, cross-sectional study of 180 participants (125 with glaucoma and 55 controls), ages 55 or older, who underwent a comprehensive clinical assessment, including vision, cognition, motor function, followed by an on-road evaluation by a trained occupational therapist. Driving errors were recorded through a dual method including: 1. An in-car trained occupational therapist 2. In-car telemetry system. The frequency and severity of errors in lane maintenance and visual scanning from the in-car telemetry will be assessed and compared between participants with varying severity of glaucoma and normal controls. In addition, we will compare the frequency and severity of errors in lane maintenance and visual scanning to those recorded by the in-car driving evaluator. RESULTS/ANTICIPATED RESULTS: We anticipate (or predict) that the in- car telemetry system will be able to detect frequency and severity of driving errors in lane maintenance and visual scanning in glaucoma participants. We also predict that participants with worsening glaucoma severity will commit more driving errors. In addition, the in-car telemetry will detect a similar frequency and severity of driving errors as the in-car driving evaluator. DISCUSSION/SIGNIFICANCE OF FINDINGS: The type and frequency of vision-related driving errors that place individuals at risk for a car accident is not well known. Without this critical information, it is extremely challenging to help older adults with glaucoma to be safe drivers.

